# Low Potency Homeopathic Remedies and Allopathic Herbal Medicines: Is There an Overlap?

**DOI:** 10.1371/journal.pone.0074181

**Published:** 2013-09-03

**Authors:** Dezső Csupor, Klára Boros, Judit Hohmann

**Affiliations:** Department of Pharmacognosy, University of Szeged, Szeged, Hungary; Royal College of Surgeons, Ireland

## Abstract

Classical homeopathy is based on the therapeutic application of highly diluted homeopathic stocks. The indications of such medicines are determined by proving, i.e. by applying the remedies in healthy subjects. However, there are several complex homeopathic medicinal products on the market with approved therapeutic indications. The efficacy of these medicines has been assessed in clinical trials on patients. There is no upper limit of dosing for such homeopathic remedies, and these products often contain undiluted mother tincture. The aim of our study was to compare an allopathic herbal medicine and a homeopathic product containing undiluted mother tincture based on the same plant. Two products (an allopathic herbal medicine and a homeopathic product) containing *Vitex agnus-castus* extract were analyzed by HPLC-DAD for their agnuside and casticin contents. The agnuside content of the allopathic product was approximately four times higher, while the amount of casticin was in the same order of magnitude. Our experiments revealed the presence of active ingredients in allopathic quantity in a homeopathic preparation, highlighting the controversy between the principles of classical and practice of contemporary homeopathy. According to the principles of classical homeopathy these remedies cannot be considered as homeopathic remedies but rather as (allopathic) herbal ones. This phenomenon necessitates a case-by-case approach towards the possible adverse effects and drug interactions of homeopathics in the daily medical practice. Homeopathic products containing active agents in allopathic doses should be treated the same way as allopathic medicines from the point of view of quality assurance and pharmacovigilance.

## Introduction

Homeopathy, an alternative medicinal method developed by Samuel Hahnemann in the 19^th^ century, is based on the Law of Similars. According to this, an appropriate homeopathic medicine contains a reduced dose of a substance which causes symptoms in a healthy person when administered in a larger quantity. The homeopathic medicinal product stimulates the self-healing mechanisms and the body’s defense system against such symptoms.

Hahnemann was beginning to use ever decreasing amounts of his preparations, and believed in the power of ‘simplexes’ or individual medicines, rather than of complex mixtures. Although classical homeopaths still adhere to Hahnemann’s principles in prescribing only single remedies, it has been described by some therapists that certain homeopathic remedies can be mixed and administered successfully as a complex. Homeopathic complexes are extremely popular in many countries: in the USA, almost 90% of all health food outlets keep them. In Europe, several hundreds of complex homeopathic medicines are on the market. Complex remedies have never been subjected to provings (i.e. determination of the curative power of the homeopathic product), although their constituents may have been proven individually. Therefore complex remedies cannot be administered according to the Law of Similars [1].

In European legislation the same directive covers both the classical individual and complex medicines. According to the Directive 2001/83/EC, homeopathic medicinal products are medicinal products prepared from substances called homeopathic stocks (mother tinctures) in accordance with homeopathic manufacturing procedures. The Directive distinguishes two major groups of homeopathic medicines: medicinal products with or without specific therapeutic indications. Those without specific therapeutic indications may be subject to a simplified registration procedure, without the need for confirmation of efficacy. The registration procedure of homeopathic medicines with therapeutic indications is the same as that of allopathic medicinal products, i.e. their safety and efficacy should be proved. Homeopathic remedies undergoing a simplified registration procedure may not contain either more than one part per 10 000 of the mother tincture or more than 1/100^th^ of the smallest dose used in allopathy with regard to active substances whose presence in an allopathic medicinal product results in the obligation to submit a doctor’s prescription. However, for homeopathic remedies with specific therapeutic indications there is no upper limit of dosing [2].

In Europe, the majority of complex homeopathic medicines are registered with approved therapeutic indications. About 65% of all remedies are prepared from extracts of plant materials [1]. Several complex remedies contain herbal extracts of D1–D4 potencies (i.e. a 1 in 10 dilution carried out serially 1–4 times) or even the undiluted mother tincture.

Though the analytical assessment of homeopathic preparations is a novel approach, modern analytical methods allow the qualitative and quantitative assessment of highly diluted homeopathic products [3], [4]. The aim of the present work was to carry out a qualitative and a quantitative comparative analysis of a herb-based homeopathic medicine and an authorized medicine based on the same medicinal plant as a reference. For this purpose, the homeopathic medicine Mastodynon tablets and the allopathic medicine Agnucaston coated tablets (both are products of Bionorica AG, Germany) both containing *Vitex agnus-castus* L. (chaste tree) fruit extract were analyzed. Both products have been approved in several European countries. Two tablets of Mastodynon (daily dosage) contain the dry matter of 324 mg *Vitex agnus castus* mother tincture, 162 mg *Caulophyllum thalictroides* D4, 162 mg *Cyclamen europaeum* D4, 162 mg *Strychnos ignatii* D6, 324 mg *Iris versicolor* D2, 162 mg *Lilium lancifolium* D3 [5]. The daily dose of Agnucaston contains 3.2–4.8 mg Agni casti fructus extract (drug-extract ratio 8.3–12.51∶1, extracting solvent 70% (V/V) ethanol) [6]. From a materialistic point of view, the main constituent of both medicines is chasteberry. The mother tincture of *Vitex agnus-castus* is produced by maceration or percolation from 1 part freshly powdered dried ripe fruits and 10 parts 62% m/m (67.4% V/V) alcohol [7]. Thus, the daily dose of the homeopathic medicine contains chasteberry extract equivalent to 32.4 mg plant material, which is similar to that of the allopathic product (43.3 mg), and the polarity of the extracting solvent applied in case of the latter is also very similar (70% V/V alcohol).

The indications of the two products are similar (premenstrual symptoms and irregular menstrual cycle for Mastodynon; [5] menstrual disturbances, mastodynia and premenstrual syndrome (PMS) for Agnucaston [6]), which is not usual for allopathic and homeopathic medicines containing the extract of the same plant. However, the listed indications are in line with the clinical evidence for chasteberry. The efficacy of *Vitex* in PMS is extensively investigated. In a prospective, randomized, placebo-controlled study with 178 participants a 60% ethanol extract of chasteberry corresponding to 180 mg dry plant material per day on average reduced the symptoms of PMS by the end of the third menstrual cycle compared to placebo [8]. In an observational study, 428 women with PMS were treated with a *Vitex* extract (solvent 60% ethanol) corresponding to 360 mg plant material. After three months, 63.3% of the physicians claimed that the three typical symptoms of PMS from which their female patients suffered the most were treated successfully [9]. The efficacy of *Vitex* has been demonstrated even in a much lower posology. In a randomized, prospective, double-blind, placebo-controlled, parallel-group, multicenter study with 217 participants, patients received 4 mg of dried 70% ethanolic extract of chasteberry (corresponding to 40 mg of plant material) once daily or placebo throughout three cycles. The symptoms of premenstrual syndrome decreased significantly in the actively treated group compared to placebo [10].

The efficacy of homeopathic chasteberry products in PMS has also been confirmed. In a double-placebo study with 104 participants Mastodynon tablets and solution were assessed for their effectiveness in relieving mastalgia. At the end of three menstrual cycles, the intensity of breast pain was significantly lower for both pharmaceutical forms compared to placebo [11]. In a double-blind study with 97 participants the intensity of mastalgia was reduced significantly in the group treated with Mastodynon solution compared to placebo after three menstrual cycles [12].

The main and characteristic constituents of chasteberry are flavonoids, compounds of essential oil and diterpenes. Experimental data cites the iridoid glycoside agnuside and the flavonol casticin as the two quality control markers for pharmaceutical grade manufacturing [13].

The exact mechanism of action and the active constituents of *Vitex* are unknown, however, some of its effects demonstrated *in vitro* and *in vivo* may play a role in the clinical efficacy. Premenstrual symptoms, particularly mastodynia, are often accompanied by latent hyperprolactinemia [14]. Mild D_2_-receptor-agonistic properties of *Vitex agnus-castus* have been demonstrated, resulting in suppressed prolactin release from cultivated lactotrophs as well as in animal experiments. In line with this observation, serum prolactin levels were indeed reduced in patients treated with this extract [15]. An agonistic action on µ- and potentially κ-opioid receptors may also be of relevance to the use of *Vitex* for menopause-related symptoms such as hot flushes and mood symptoms [14]. Extracts were demonstrated to displace ligands from human opioid receptor binding [13]. A relatively potent binding inhibition was observed for opioid receptors (µ and κ subtypes) with IC_50_ values of the native extract between 20 and 70 mg/ml [16]. The results of an *in vitro* study suggested that *Vitex* acts as an agonist at the µ-opioid receptor, supporting its beneficial action in PMS [17]. A 90% methanol extract of chasteberry was found to bind to and activate μ- and δ-, but not κ-opioid receptor subtypes. Several flavonoids isolated from *Vitex* were found to bind to both μ- and δ-opioid receptors in a dose-dependent manner; however only casticin was found to have an agonistic activity selective for δ receptors at high concentrations [18].

## Results

**Figure 1:**
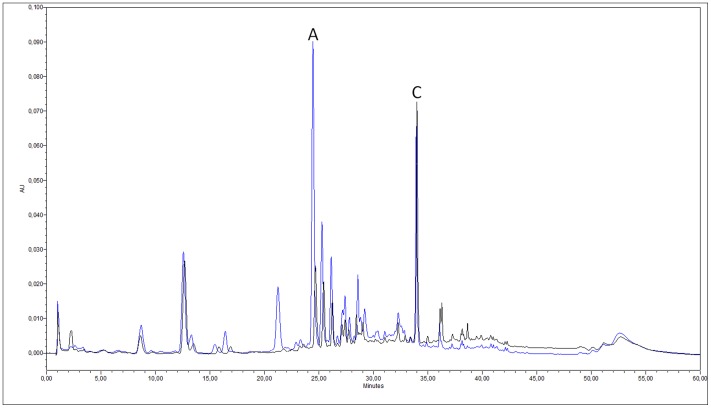
HPLC profiles of the allopathic medicine Agnucaston and the homeopathic preparation Mastodynon (blue: Agnucaston, black: Mastodynon; agnuside (A), casticin (C)).

In the course of our experiments we quantified the agnuside and casticin contents of Mastodynon tablets and Agnucaston coated tablets by HPLC–DAD (**Figure 1**). The fingerprint chromatograms of the two products were very similar. One of the main differences was that the agnuside/casticin ratio was found to be 2.35 for Agnucaston and 0.54 for Mastodynon. The average agnuside content of the daily dose of Agnucaston and Mastodynon was 100.82 µg and 25.19 µg, respectively. The casticin content in the daily dosage of the two products was 42.95 µg and 47.03 µg, respectively.

### Calibration Curve and Linearity

Linear calibration curves were established in the range of 0,05–1 µg/injection for agnuside and 0,058–0,58 µg/injection for casticin. The regression equation for agnuside was y = 1 944 361,72x+27 039,23. The correlation coefficient (R^2^) was 0.9994. In case of casticin the regression equation was y = 1 916 347,45x+815,49 (R^2^ = 0.9996).

### Precision

The analytical method is characterized by good precision (RSD% for agnuside standard solution: 2.41, for casticin: 1.26). In case of the two products the repeatability of agnuside and casticin content determination ranges from 2.11 to 5.11 RSD. The maximal deviation from the average content of casticin and agnuside was between 92.16% and 109.48%, which complies with the test for uniformity of content of single-dose preparations of the European Pharmacopoeia (85%–115%).

### Limit of Detection (LOD) and Limit Of Quantitation (LOQ)

The limit of detection obtained for agnuside was 3.872 ng/injection and limit of quantitation was 12.905 ng/injection. For casticin, the same values were 1.079 ng/injection and 3.596 ng/injection, respectively.

### Peak Purity

Peak purity analysis carried out with the Empower Pro software confirmed that the chromatographic peaks of agnuside and casticin were spectrally pure based on the fact that the plot of purity angles were below the plot of threshold angles and the good accordance of the UV spectra obtained at three different retention times (at the peak maximum and +/−1/3 of the peak width). Pearson’s correlation coefficients, characterizing peak purities, were determined based on the similarities of UV spectra of the standard solutions and the peaks in the samples in the course of HPLC-DAD analysis. The correlation coefficients, which were calculated using spectra detected at the apex, the upslope and the download segments of the peaks, were higher than 0.998 in all cases.

## Discussion

Based on the chromatographic profile it is hard to distinguish between the allopathic and the homeopathic medicinal product. However, it is clear that the two products contain somewhat different chasteberry extracts since the agnuside/casticin ratios are different. The quantities of agnuside are of the same order and the amounts of casticin are practically equal in the daily doses. Though the two quantified constituents are not necessarily the active agents of *Vitex agnus-castus*, these compounds are recognized as quality control markers and provide information on the quantity of the herbal extract. The minor constituents of the homeopathic medicine were not analyzed. However, from an analytical point of view, Mastodynon is a monocomponent product when analyzed with HPLC-DAD, since other constituents are present at concentrations lower by several orders of magnitude.

## Materials and Methods

Mastodynon tablets (lot no. 0000015632) and Agnucaston coated tablets (lot no. 0000023527) (both produced by Bionorica AG, Germany) were purchased in a pharmacy in Szeged, Hungary. Agnuside (99.12% purity) was obtained from Phytolab. Casticin was isolated from plant material in the Department of Pharmacognosy, University of Szeged [19]. HPLC grade methanol (LiChrosolv®) was obtained from Merck and phosphoric acid (85%) from Reanal Fine Chemicals. A Millipore Direct Q-3 water purification system was used to produce HPLC grade deionized water.

### Preparation of the Extracts

The daily doses of the two products (1 tablet of Agnucaston, 2 tablets of Mastodynon) were sonicated for 15 min in 3 ml methanol. After 5 min centrifugation at 7500 rpm, the supernatant was filtered through an Acrodisc® GHP 13 mm, 0.45 µm filter (Waters). The first ½ ml was disposed and the remaining solution was analyzed by HPLC. From agnuside and casticin, stock solutions were prepared with methanol (0.05 and 0.029 mg/ml, respectively).

### HPLC Analysis

Extracts of the two chasteberry-containing products were characterized by HPLC (Waters 600, equipped with a 2998 photodiode array detector, on-line degasser and autosampler) using a slightly modified fingerprinting method described previously [16], [18]. Using a reversed phase Gemini C18 110 Å 100 × 4.6 mm 5 µm column (Phenomenex), chromatographic elution was accomplished by a gradient solvent system consisting of methanol (A) and 0.5% phosphoric acid in water (B). The gradient conditions were: 0 min, 5∶95 (v:v); 15 min, 27∶73; 20 min, 35∶65; 26 min, 52∶48; 33 min, 80∶20; 37 min, 100∶0; 45 min 100∶0; 50 min, 5∶95; 60 min, 5∶95. The flow rate was 1.3 ml/min, the injection volume was 20 µl. Agnuside and casticin were detected at 254 nm and identified based on their retention times and PDA chromatograms.

### Method Validation

From both products, three individual extracts were prepared and analysed.

### Calibration Curve and Linearity

The calibration curve was determined on each day of the validation; the slope, intercept and the correlation coefficient were determined. Calibration curves were established within a range of 0.005–0.05 mg/ml for agnuside and 0.0029–0.029 mg/ml for casticin by diluting the stock solutions with MeOH in appropriate quantities. The linearities of the calibration curves were assessed based on 5 points.

### Precision

To determine the precision of the analytical method, the same standard solutions and extracts were subjected to HPLC–DAD analysis 3 times on the same day. Agnuside and casticin contents of the samples were measured, and the relative standard deviation (RSD) values within the measurements were used to evaluate precision.

### Limit Of Detection (LOD) and Limit Of Quantitation (LOQ)

Limit of detection and limit of quantitation were calculated using the formulas (1) and (2), respectively:
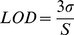
(1)

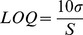
(2)where *S* is the slope of the calibration curve and 
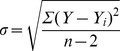
 where *Y* is the measured peak area, *Y_i_* is the peak area calculated by the calibration curve, and *n* is the number of the calibration points.

### Peak Purity

Peak purity analysis was carried out with the Empower Pro software.

## Conclusions

As highlighted by our experiments, homeopathy is not a uniform medicinal system, and homeopathic remedies can be grouped into at least two major classes: remedies of high potency (extremely high dilution and low dose) and those of low potency or even mother tinctures (doses near or equal to allopathic doses). In case of the latter group, the principles of Hahnemann are not applicable. Low dilutions and mother tinctures play a role in homeopathic prescribing, and are particularly prominent in systems of homeopathy focusing on the organotropic effects of homeopathic medicinal products [20]. In the European Union, the Directive 2001/83/EC [2] allows the authorization of homeopathic medicines containing allopathic doses, thus the distinction between low potency homeopathic medications and allopathic herbal medicines may be based on the intention of the manufacturer, i.e. on the production method and the rationale behinds their application and not on the dose. Our results reassure and highlight that homeopathic products may contain ingredients in allopathic doses. According to the principles of classical homeopathy these remedies cannot be considered as homeopathic remedies but rather as (allopathic) herbal ones. It is arguable whether products containing herbal extracts with pharmacologically relevant doses meet the health professionals’ and patients’ expectations for homeopathy. Since these preparations should be treated the same way as allopathic medicines from the point of view of pharmacovigilance, health professionals should be aware of this very special subgroup of homeopathics.
